# INS/LIDAR/Stereo SLAM Integration for Precision Navigation in GNSS-Denied Environments

**DOI:** 10.3390/s23177424

**Published:** 2023-08-25

**Authors:** Nader Abdelaziz, Ahmed El-Rabbany

**Affiliations:** 1 Department of Civil Engineering, Toronto Metropolitan University, Toronto, ON M5B 2K3, Canada; rabbany@torontomu.ca; 2 Department of Civil Engineering, Tanta University, Tanta 31527, Egypt

**Keywords:** INS/LiDAR/stereo SLAM, ORB-SLAM, integrated navigation system, GNSS-denied environments, redundant navigation system

## Abstract

Traditionally, navigation systems have relied solely on global navigation satellite system (GNSS)/inertial navigation system (INS) integration. When a temporal loss of GNSS signal lock is encountered, these systems would rely on INS, which can sustain short bursts of outages, albeit drift significantly in prolonged outages. In this study, an extended Kalman filter (EKF) is proposed to develop an integrated INS/LiDAR/Stereo simultaneous localization and mapping (SLAM) navigation system. The first update stage of the filter integrates the INS with the LiDAR, after which the resultant navigation solution is integrated with the stereo SLAM solution, which yields the final integrated navigation solution. The system was tested for different driving scenarios in urban and rural environments using the raw Karlsruhe Institute of Technology and Toyota Technological Institute (KITTI) dataset in the complete absence of the GNSS signal. In addition, the selected KITTI drives covered low and high driving speeds in feature-rich and feature-poor environments. It is shown that the proposed INS/LiDAR/Stereo SLAM navigation system yielded better position estimations in comparison to using the INS without any assistance from onboard sensors. The accuracy improvement was expressed as a reduction of the root-mean-square error (RMSE) by 83% and 82% in the horizontal and up directions, respectively. In addition, the proposed system outperformed the positioning accuracy of some of the state-of-the-art algorithms.

## 1. Introduction

Precise positioning is one significant obstacle to overcome to achieve accurate vehicular navigation. An effective navigation system should be capable of functioning effectively in any driving scenario, such as in urban or rural areas with high or low traffic, and in varying weather and lighting conditions. Moreover, incorporating redundant sensors is a vital aspect to consider when creating a navigation system, which formulates a robust navigation system. For a navigation system to function securely and accurately, it is necessary to have multiple onboard sensors, which ensures that if one sensor fails, for any reason, the other sensors can compensate for the malfunction, and thereby the system continues to operate safely and effectively [1,2,3].

The integration of the global navigation satellite system (GNSS) with the inertial navigation system (INS) has been extensively researched and implemented in numerous studies [4,5,6,7,8,9,10,11]. Typically, the GNSS and the inertial measurement unit (IMU) measurements are merged using a Kalman filter [12], which can employ various integration methods between the GNSS and the IMU, such as loosely coupled and tightly coupled integrations. Loosely coupled integration of GNSS/INS involves combining independent outputs of both systems to enhance navigation accuracy and reliability, while tightly coupled integration integrates GNSS measurements directly into INS computations [13,14]. However, relying solely on INS in the event of prolonged GNSS signal outages can lead to degraded positioning accuracy, especially when utilizing a low-cost micro-electro-mechanical system (MEMS) IMU. As a result, it is crucial to mount more onboard sensors, such as camera(s) or LiDAR sensors.

Using a camera or a LiDAR sensor for positioning operates on simultaneous localization and mapping (SLAM) algorithms. SLAM can be formally defined as the ability to map the surroundings while simultaneously monitoring the sensor’s location [15]. Many visual SLAM (VSLAM) algorithms were proposed as collected in [16]. Visual SLAM employs visual markers or landmarks to establish a map of the surroundings and determine the sensor’s position. Typically, visual SLAM utilizes a camera as the primary sensor and analyzes images to identify environmental features. By examining the motion of these features between successive images, visual SLAM can approximate the sensor’s location while generating a map of the area. Visual SLAM can be executed using various types of cameras, including monocular cameras [17,18,19,20,21], which retrieve the trajectory up to a scale factor, and stereo cameras [22,23,24], which correct the scale factor. It is worth mentioning that visual SLAM has the key benefit of capturing considerably more environmental details compared to using LiDAR. However, contrary to LiDAR SLAM, visual SLAM is considerably impacted by variations in illumination. It is worth mentioning that in some lighting conditions (i.e., bright sunlight), the LiDAR sensor performance can be negatively affected as a result of interference or backscattering from highly reflective targets [25].

Similar to the developed visual SLAM algorithms, a large number of studies proposed many LiDAR SLAM algorithms, the leading of which is the LiDAR odometry and mapping (LOAM) [26] which was tested on the KITTI odometry benchmark [27]. As a result, numerous variations of LOAM, including A-LOAM and Kitware [28,29,30,31], have been created, which have enhanced the computational efficiency of the original LOAM algorithm. It is worth mentioning that a major advantage of LiDAR SLAM is that it is not impacted by lighting conditions because the LiDAR scanner is an active sensor.

Considering the advantages of both LiDAR sensors and cameras, the most ideal scenario would be to integrate both sensors (i.e., the camera and LiDAR) to obtain rich environmental information while reducing any negative effects of illumination variations.

Several studies have incorporated an INS with either a LiDAR sensor or a camera. In our previous work in [32], we introduced a loosely-coupled integrated navigation system, which aimed to combine INS and LiDAR SLAM using an EKF. The efficacy of this novel navigation approach was evaluated across a range of driving scenarios and environments, utilizing the raw residential and highway datasets from the KITTI dataset. The study comprised three distinct case studies. The first case study considered residential datasets from the KITTI collection, encompassing a total driving duration of 48 min during a complete GNSS signal outage. The second case study delved into highway datasets spanning a total duration of 7 min, encompassing all available highway datasets within the raw KITTI data. Paralleling the first case study, the LiDAR SLAM system showcased better positional results in extended datasets. The third case study tackled scenarios involving intermittent GNSS signal outages, simulating situations like urban canyons and tunnels on highways. The results demonstrated significant enhancements when compared to the same datasets in the first case study, which dealt with complete GNSS signal loss. Across all driving scenarios, the integrated INS/LiDAR SLAM navigation system showcased substantial improvements in positional accuracy for residential datasets, achieving an average RMSE reduction of 88% and 32% in the horizontal and vertical components, respectively. The improvements for highway datasets were approximately 70% and 0.2% for the horizontal and vertical components, respectively. Comparisons were drawn between the proposed system and three state-of-the-art LiDAR SLAM algorithms. While the system slightly outperformed its counterparts for residential datasets, it significantly outshone other SLAM algorithms in highway environments. This comprehensive investigation underscores the potential of the proposed integrated navigation system across various driving scenarios and its notable contributions to enhancing navigation accuracy and reliability.

Similarly, another study [33] integrated INS and LiDAR SLAM in an unmanned aerial vehicle (UAV) mapping system to address a malfunctioning GNSS, and the developed GNSS/INS/LiDAR SLAM system was able to overcome GNSS outages. In another research [34], an EKF was used to fuse data from 3D-RISS, GNSS, and LiDAR odometry (LO) to improve positioning accuracy, yielding a 64% decrease in positioning errors compared to using the INS only. In [35], a navigation model that integrated a monocular camera, IMU, and GNSS to navigate ground vehicles in GNSS environments that are susceptible to signal outages was proposed. The system yielded up to a 74% reduction in position error compared to using only GNSS data.

In this research, we extend our work in [32] by proposing an integration between the INS, LiDAR SLAM, and stereo visual SLAM, which leverages the often overlooked capabilities of the INS as a navigation sensor that can produce reliable information. Additionally, the developed navigation model stands out in terms of achieving redundancy, which is crucial for safe and efficient navigation. The integration is executed using a series of EKFs in a loosely coupled integration scheme. To comprehensively validate the performance of our advanced navigation model, we conducted extensive testing across various drives utilizing raw data from the KITTI datasets. These drives encompassed a wide spectrum of driving scenarios, ranging from intricate urban environments to sprawling rural landscapes, and included variations in driving speeds. Finally, our integrated system is weighed against some state-of-the-art models.

## 2. Navigation System Architecture

### 2.1. LiDAR SLAM

The Kitware SLAM [28], an updated version of the LOAM algorithm [26], is the LiDAR SLAM employed in this study. In our work in [32], a thorough explanation of Kitware SLAM and the various improvements it offers over LOAM is presented.

### 2.2. Stereo Visual SLAM

ORB-SLAM [21,24,36] can be considered a superior state-of-the-art visual SLAM algorithm that can be implemented for both monocular and stereo cameras. Therefore, ORB-SLAM3 [36,37] was adopted in this research to process the stereo images of the KITTI data.

The pipeline of the stereo SLAM system consists of feature detection, feature matching, pose estimation, point cloud construction, and bundle adjustment. Using a scale-invariant feature transform (SIFT) algorithm, ORB features are detected and matched in the left and right stereo images, followed by orientation estimation using the intensity centroid method. The matched points are utilized to calculate the fundamental matrix, and the relative camera pose between frames is then estimated by solving the five-point algorithm with RANSAC to exclude outliers. Using a robust optimizer known as the robust information matrix optimization (RIMO) algorithm, which takes into account the feature measurements’ uncertainty, the camera pose estimation is further refined. The environment’s 3D point cloud is generated by triangulating the matched feature points in the left and right camera images using relative pose estimates. Using bundle adjustment, the point cloud is optimized to minimize the reprojection error of the 3D points onto the stereo images. Utilizing the Levenberg–Marquardt algorithm with the Schur complement factorization, the optimization is carried out. The optimization of bundle adjustment includes robust loss functions to manage outliers and noise. Using a bag-of-words approach, loop closures are identified by comparing the current image to a database of prior images. The method represents images using histograms of visual words and matches the current image to the database entry with the highest degree of similarity. Using geometric constraints, the loop closure is validated, and then the map and camera configurations are optimized using bundle adjustment.

### 2.3. Integrated Navigation Systems

#### 2.3.1. Coordinate Transformations

**Figure 1:**
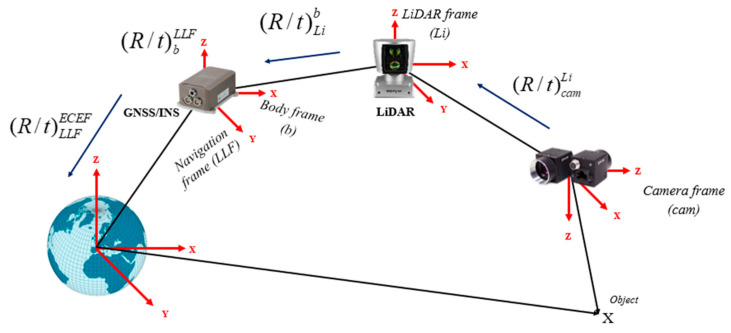
Pose transformation of onboard sensor WGS84 global reference frame.

SLAM pose estimations are computed relative to the sensor’s local reference frame. In other words, all poses in LiDAR SLAM are in reference to the first LiDAR frame, while in Visual SLAM, they are based on the first camera frame. Therefore, all local coordinates will be transformed from local frames to the WGS84 ellipsoid, which is necessary for the subsequent Kalman filtering. Figure 1 illustrates the process of transformation graphically.

Pose transformations are executed using 4 × 4 homogenous transformation matrices. Let $P_{i}{}^{cam}$ and $P_{i}{}^{Li}$ denote the coordinates of a point expressed in the camera frame and LiDAR frame, respectively. Let $P_{i}{}^{ecef}$ denote the same point expressed in the WGS84 reference frame. The same point referenced to the WGS84 reference frame is denoted by $P_{i}{}^{ecef}$. Equations (1)–(4) represent the sequence of homogeneous transformations.

(1) $$P_{i}{}^{ecef}={\left(R/t\right)}_{l}^{ecef}{\left(R/t\right)}_{b}^{l}{\left(R/t\right)}_{Li}^{b}{\left(R/t\right)}_{cam}^{Li}P_{i}{}^{cam}$$

(2) $$P_{i}{}^{ecef}={\left(R/t\right)}_{l}^{ecef}{\left(R/t\right)}_{b}^{l}{\left(R/t\right)}_{Li}^{b}P_{i}{}^{Li}$$

(3) $$R_{cam}^{l}=R_{b}^{l}R_{Li}^{b}R_{cam}^{Li}$$

(4) $$R_{Li}^{l}=R_{b}^{l}R_{Li}^{b}$$

where ${\left(R/t\right)}_{subscript-origin}^{superscript-destination}$ symbolizes the 4 × 4 homogeneous transformation matrix from the origin frame to the destination frame. That is, ${\left(R/t\right)}_{cam}^{Li}$ is the homogenous transformation from the camera frame to the LiDAR frame, ${\left(R/t\right)}_{Li}^{b}$ denotes the homogenous transformation from the LiDAR frame to the body frame, ${\left(R/t\right)}_{b}^{l}$ is the transformation from the body frame to the local-level frame, and ${\left(R/t\right)}_{l}^{ecef}$ is the transformation from the local-level frame to the WGS84 reference frame.

#### 2.3.2. INS/LiDAR/Stereo SLAM Integration

**Figure 2:**
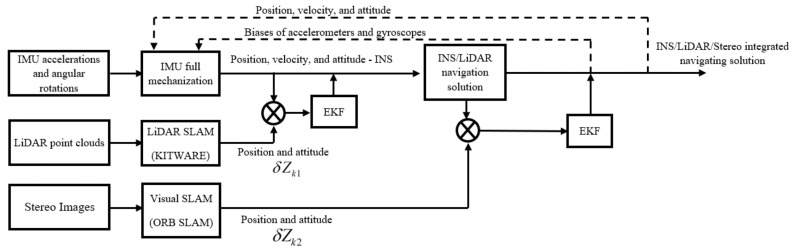
INS/LiDAR/Stereo LC integration block diagram.

The measurements of the IMU, LiDAR, and the stereo camera are fused using the proposed integrated navigation system through a series of EKFs as illustrated by the block diagram in Figure 2. Firstly, the INS solution is fused with the LiDAR SLAM pose estimations through the first EKF, forming the navigation solution of the INS/LiDAR SLAM integration. The latter is then fused with the stereo SLAM solution in the second EKF to form the final integrated INS/LiDAR/Stereo navigation system. Finally, the updated errors are fed back into the IMU’s full mechanization, which creates a closed-loop error scheme.

The EKF mathematical and stochastic models (i.e., the system model, the design matrix (H), and the system dynamic matrix (F)) are the same as our work in [32]. The state vector is given by Equation (5). The first and second update vectors are given by Equations (6) and (7), respectively.

(5) $$\delta x={\left[\begin{array}{ccccc} \delta r & \delta v & \delta \varepsilon & \delta b_{a} & \delta b_{g} \end{array}\right]}^{T}$$

(6) $$\delta Z_{k1}={\left[\begin{array}{cc} \delta r_{1} & \delta \varepsilon_{1} \end{array}\right]}^{T}={\left[\begin{array}{cccccc} \varphi_{INS-Li} & \lambda_{INS-Li} & h_{INS-Li} & p_{INS-Li} & r_{INS-Li} & y_{INS-Li} \end{array}\right]}^{T}$$

(7) $$\delta Z_{k2}={\left[\begin{array}{cc} \delta r_{2} & \delta \varepsilon_{2} \end{array}\right]}^{T}={\left[\begin{array}{cccccc} \varphi_{NS/Li-stereo} & \lambda_{NS/Li-stereo} & h_{INS/Li-stereo} & p_{INS/Li-stereo} & r_{INS/Li-stereo} & y_{INS/Li-stereo} \end{array}\right]}^{T}$$

where $\delta r={\left[\begin{array}{ccc} \delta \phi & \delta \lambda & \delta h \end{array}\right]}^{T}$ is the error vector of the position; $\delta v={\left[\begin{array}{ccc} \delta v_{e} & \delta v_{n} & \delta v_{u} \end{array}\right]}^{T}$ is the error vector of the velocity, $\delta \varepsilon ={\left[\begin{array}{ccc} \delta p & \delta r & \delta y \end{array}\right]}^{T}$ is the error vector of the attitude; $\delta b_{a}={\left[\begin{array}{ccc} \delta b_{ax} & \delta b_{ay} & \delta b_{az} \end{array}\right]}^{T}$ is the error vector of the accelerometer bias; $\delta b_{g}={\left[\begin{array}{ccc} \delta b_{gx} & \delta b_{gy} & \delta b_{gz} \end{array}\right]}^{T}$ is the error vector of the gyroscope bias; $\delta Z_{k1}$, $\delta Z_{k2}$ are the measurement update vectors; $\delta r_{1}$, $\delta r_{2}$ are the position error vectors in the first and second update stages, respectively (i.e., latitude error: $\varphi_{INS-Li}=\varphi_{INS}-\varphi_{Li}$); and $\delta \varepsilon_{1}$, $\delta \varepsilon_{2}$ are the attitude error vectors in both update stages.

## 3. Data Acquisition and Description

In this research, the Karlsruhe Institute of Technology and Toyota Technological Institute (KITTI) dataset [38] is adopted as the data source, which is available through [39]. The KITTI dataset stands out as a valuable resource for researchers internationally and they can publish their results on the KITTI odometry benchmark [27] to enable fair comparisons between different algorithms and methods. It is renowned for its diverse sensor suite, which enables capturing data from various perspectives. The platform’s sensor configuration includes an integrated GNSS/IMU unit (OXTS RT3003), a 360-degree rotating mechanical LiDAR with 64 beams (Velodyne HDL-64E), and two Sony stereo pairs that collect both color and grayscale images. These sensors work in harmony to provide a rich and comprehensive view of the environment surrounding the data collection vehicle.

The KITTI dataset was gathered in Germany, specifically in the town of Karlsruhe. The choice of location offers an advantage for researchers, as it presents a wide range of real-world driving scenarios and environmental conditions. The dataset encompasses urban, rural, and highway scenes, making it applicable to various driving applications worldwide.

The KITTI dataset is comprised of several categories, namely raw data, object detection and tracking, road segmentation, and odometry. Each category serves a specific purpose for vehicular navigation and autonomous driving research. The raw data consists of raw sensor measurements, including images, point clouds, and sensor calibration parameters. Researchers can utilize these data to develop and test their algorithms for tasks such as object detection, semantic segmentation, and depth estimation. It is worth noting that the raw datasets are available in time unsynchronized and synchronized data, where the latter is widely utilized. The object detection and tracking dataset focuses on providing annotated 2D and 3D bounding box information for objects of interest, such as cars, pedestrians, and cyclists. It allows researchers to train and evaluate object detection and tracking algorithms. The third category of data, road segmentation, is designed to tackle road scene understanding. It provides pixel-level annotations for road regions, enabling the development of algorithms for road segmentation, a crucial task for autonomous navigation. Finally, the odometry dataset offers precise ego-motion estimates, allowing researchers to evaluate and refine their localization and mapping algorithms. It consists of synchronized camera-LIDAR sequences with accurate ground truth pose information. However, it does not contain raw IMU measurements (i.e., raw accelerations and angular rotations). Therefore, in order to take advantage of the IMU’s raw measurements which are essential components of the proposed navigation models, the raw dataset is the one considered in this study.

The structure of the KITTI raw dataset is a well-defined, consistent structure across all drives. Each dataset is organized into sequences, where each sequence represents a continuous driving segment. For instance, a sequence might cover a specific urban area or a highway stretch. Each sequence contains multiple data modalities, such as grayscale and color images, Velodyne point clouds, and GPS/IMU measurements. Additionally, the dataset includes calibration files for sensor alignment and synchronization purposes.

In this research, both residential and highway datasets are considered, which forms two case studies. The drive number of each of the raw KITTI datasets is presented in Table 1. In addition, the table presents the length, the elapsed drive time, average speed, and the number of frames of each dataset.

## 4. Analysis and Results

### 4.1. Residential Datasets—Case Study 1

**Figure 3:**
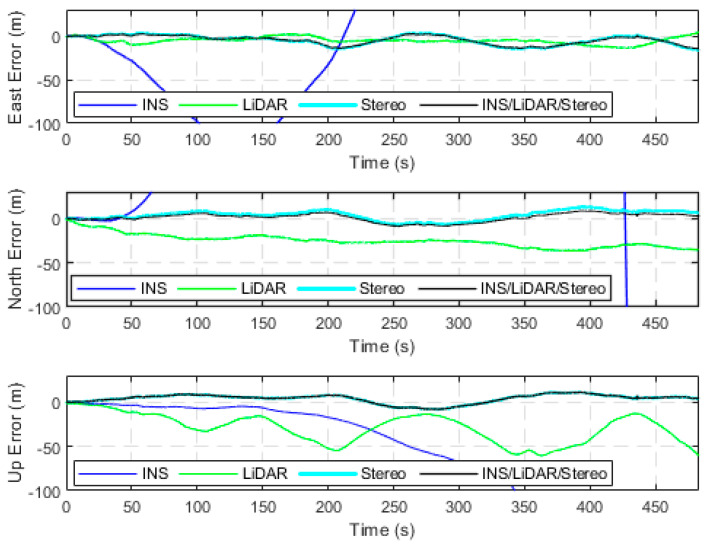
Positional errors expressed in the ENU frame, D-34.

**Figure 4:**
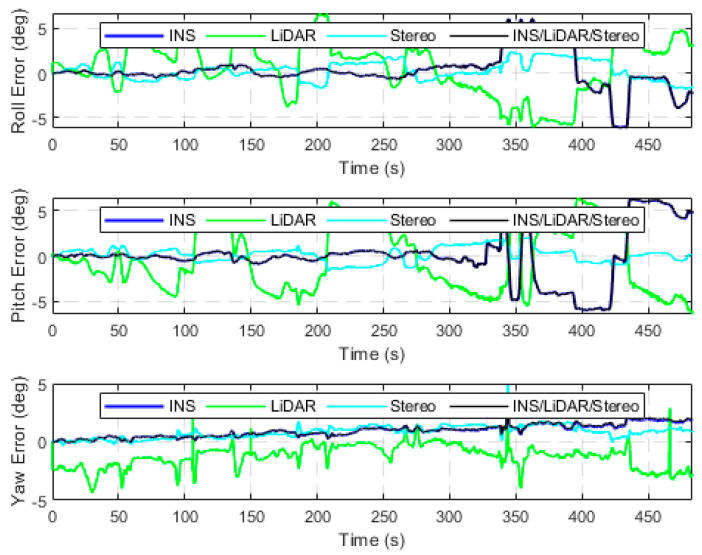
Attitude angles’ errors, D-34.

The first case study analyzes seven drives of the raw KITTI dataset, in which the driving environment is in urban areas at low speeds with high-density features. All seven datasets add up to approximately 17 kilometers of driving distance for a total driving time of around 34 min. In addition, all drives were used to test the performance of the developed integrated navigation system in residential driving environments. The first residential drive to consider was D-34, which is a 920-m drive segment averaging a speed of around 26 km/h. Figure 3 presents the position error in the ENU frame (navigation frame) for four navigation solutions, namely the INS, the LiDAR SLAM, the Stereo SLAM, and the final integrated navigation solution. These errors are calculated in reference to the reference trajectory provided by the integrated GNSS/INS unit (OXTS). The errors of the attitude angles (roll, pitch and yaw) are shown in Figure 4.

**Figure 5:**
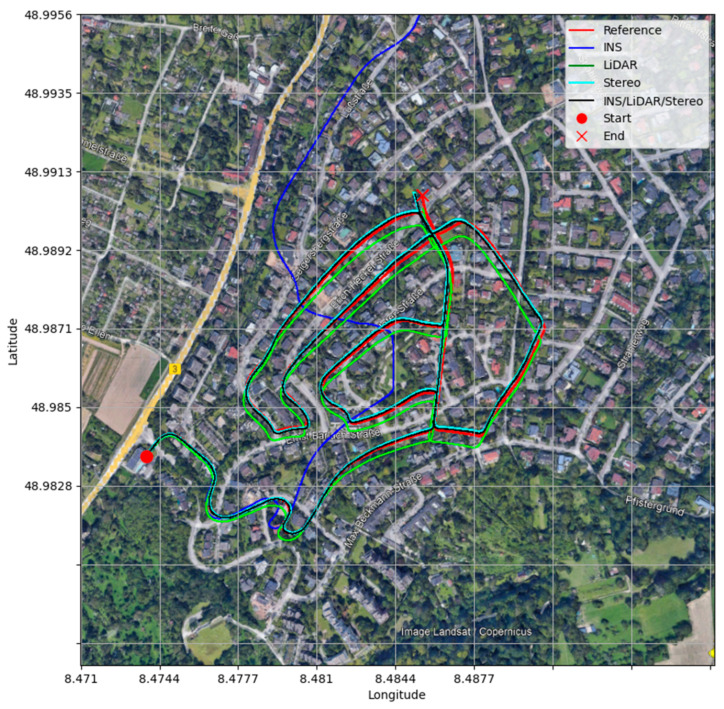
Comparison of trajectories in the world frame (WGS84), D-34, base map captured from Google Earth [40].

From Figure 3, it is observable that the INS solution drifts significantly shortly after the vehicle starts the drive, which is typical behaviour of the sole use of an IMU. Both the LiDAR and Stereo SLAM yield significantly more accurate results when compared to the INS, with the Stereo SLAM being the most accurate. Consequently, the final navigation solution followed that of Stereo SLAM for positioning results. On the contrary, in Figure 4, the INS produces more accurate and stable estimations of the attitude angles as opposed to the LiDAR and Stereo SLAM estimations. This is a result of the high-quality IMU utilized by the KITTI data collection platform. In addition, this behaviour is consistent with our previous findings in [32]. Consequently, the integrated system was tuned to match the INS’s attitude estimation. In Table 2, the error statistics of the INS/LiDAR/Stereo navigation solution are displayed, while Figure 5 compares all four trajectories to the ground truth.

**Figure 6:**
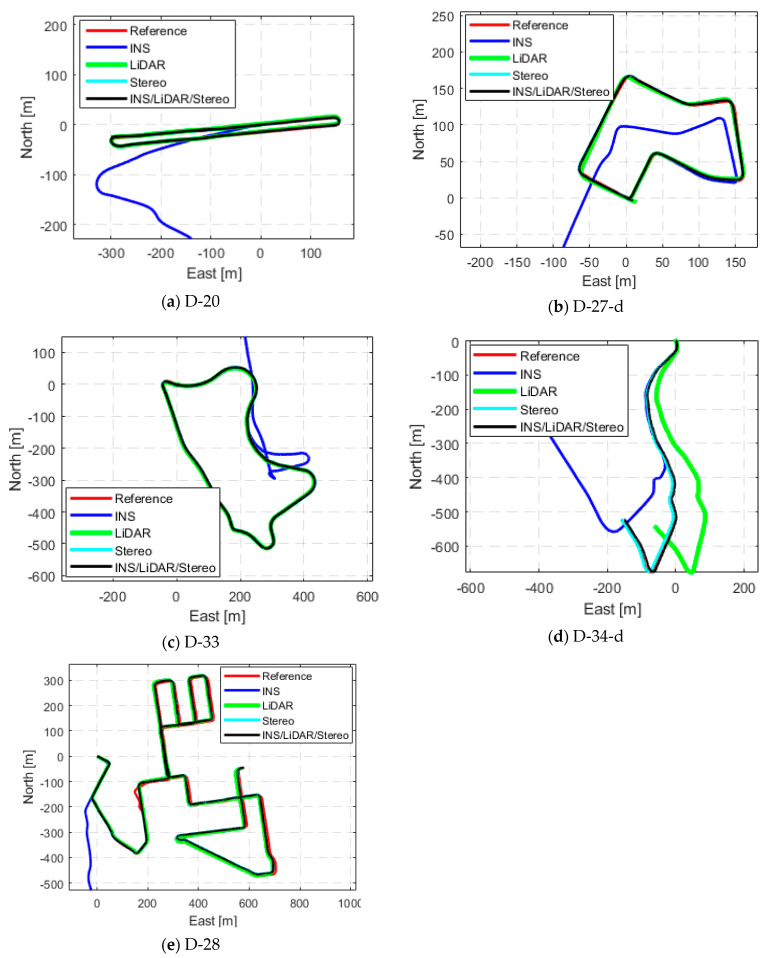
Comparison of trajectories of the raw residential KITTI drives in the ENU frame.

**Figure A1:**
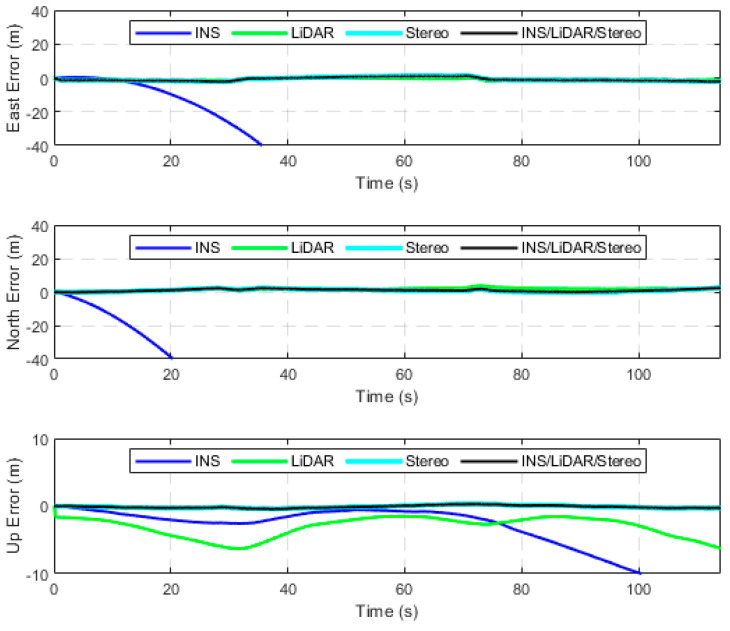
Positional errors expressed in the ENU frame, D-20.

**Figure A2:**
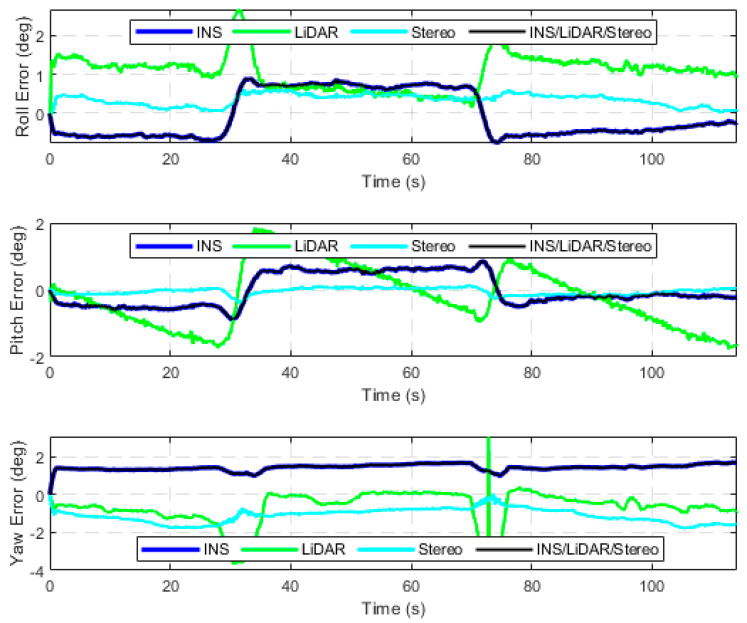
Attitude angles’ errors, D-20.

**Figure A3:**
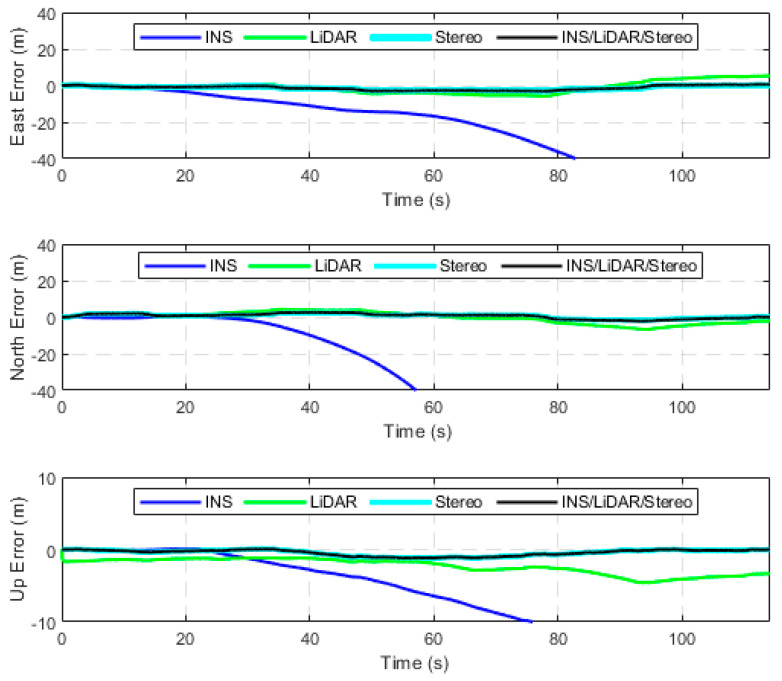
Positional errors expressed in the ENU frame, D-27-d.

**Figure A4:**
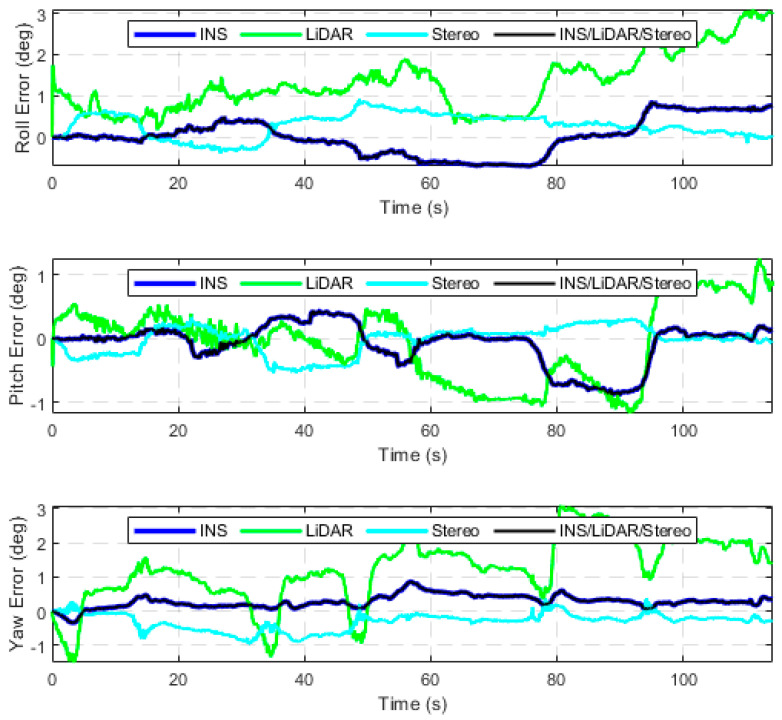
Attitude angles’ errors, D-27-d.

**Figure A5:**
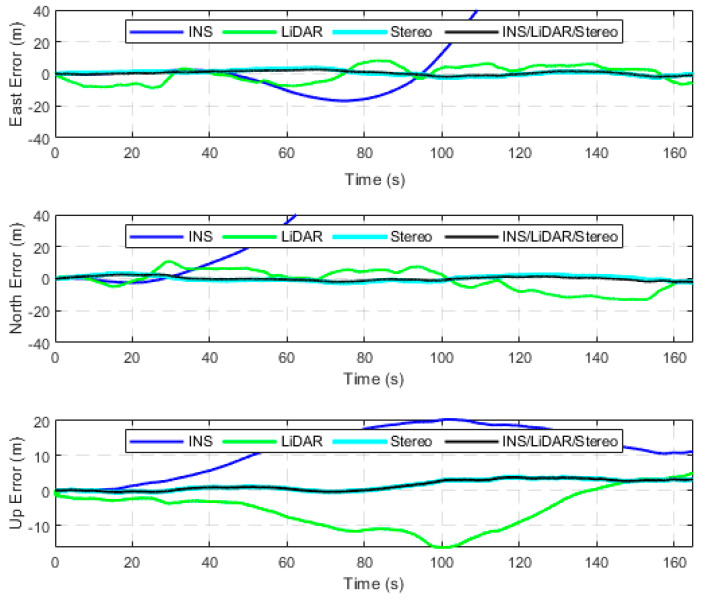
Positional errors expressed in the ENU frame, D-33.

**Figure A6:**
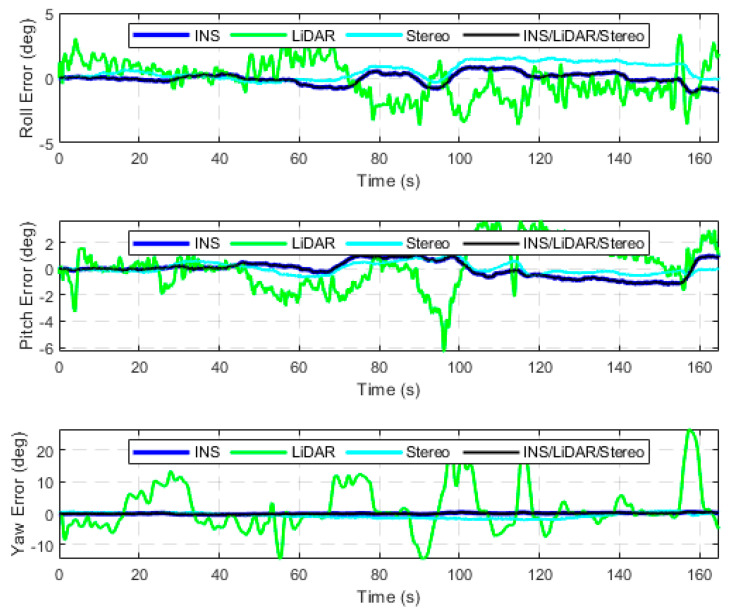
Attitude angles’ errors, D-33.

**Figure A7:**
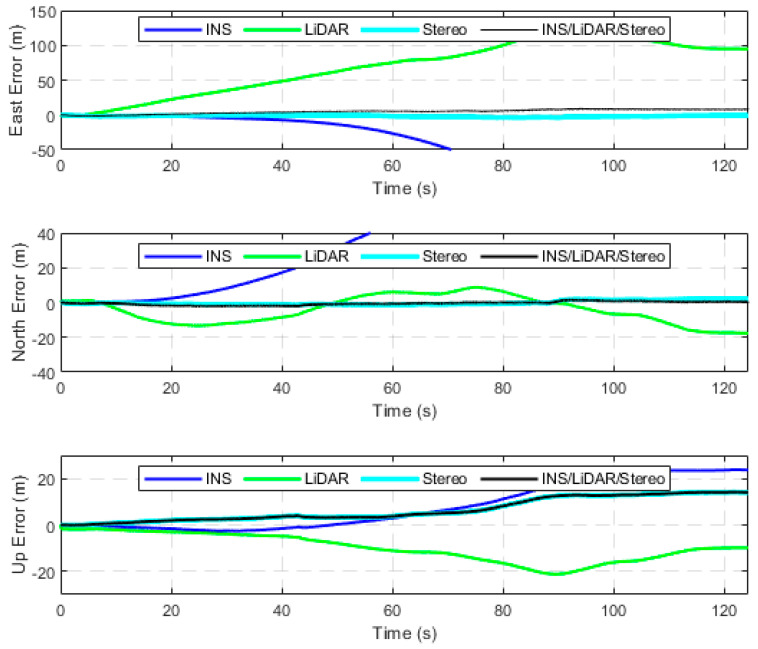
Positional errors expressed in the ENU frame, D-34-d.

**Figure A8:**
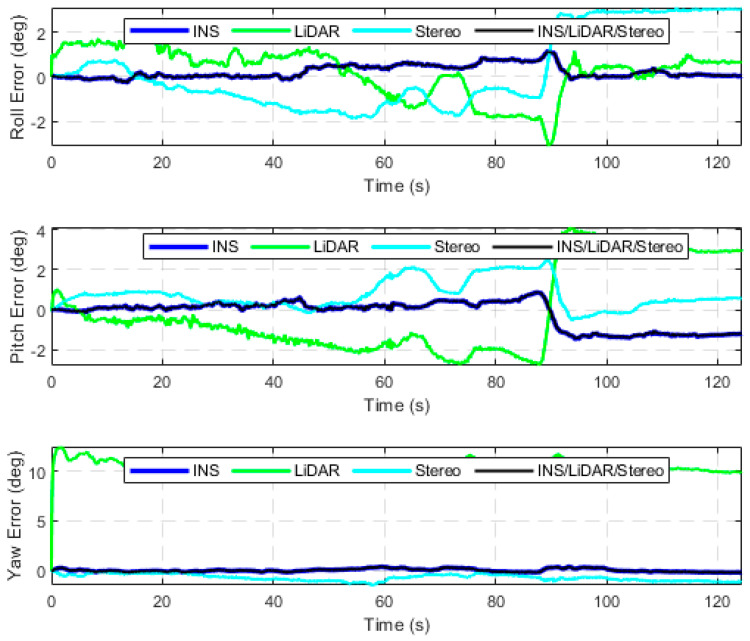
Attitude angles’ errors, D-34-d.

**Figure A9:**
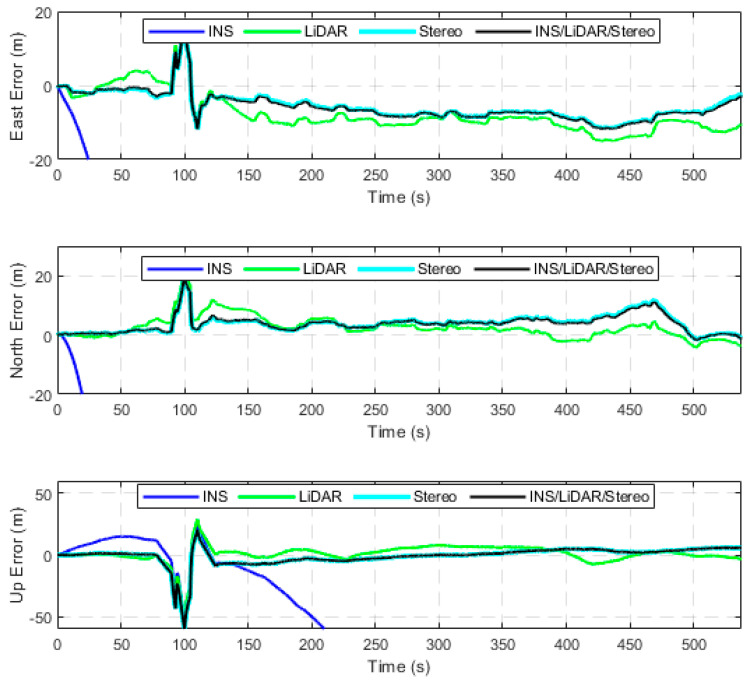
Positional errors expressed in the ENU frame, D-28.

**Figure A10:**
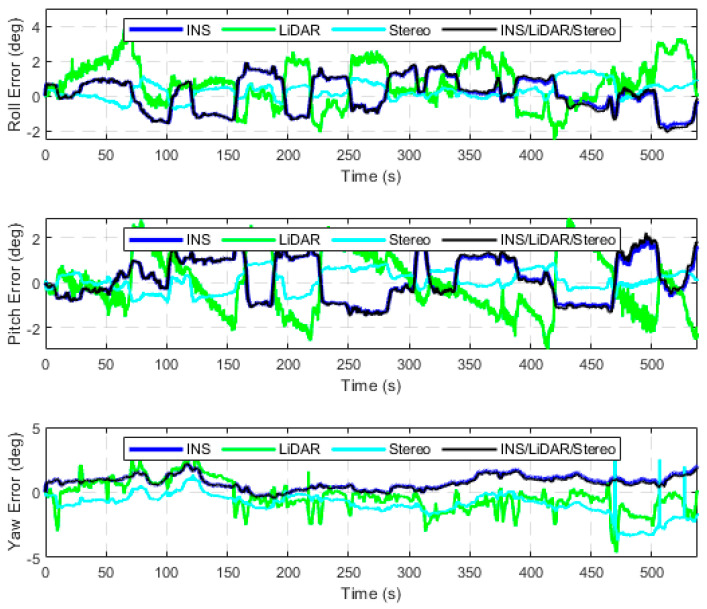
Attitude angles’ errors, D-28.

The remaining datasets in Table 1 experienced the same analysis as previously done for D-34. The datasets yielded the same trend for the position and attitude estimations. Appendix A, Figure A1, Figure A2, Figure A3, Figure A4, Figure A5, Figure A6, Figure A7, Figure A8, Figure A9 and Figure A10 provide detailed position and attitude results for each of the remaining drives of the KITTI dataset. Figure 6 compares the INS, LiDAR SLAM, Stereo SLAM, and the INS/LiDAR/Stereo integrated navigation solution to the ground truth trajectory for each drive, as displayed in the navigation frame.

**Figure 7:**
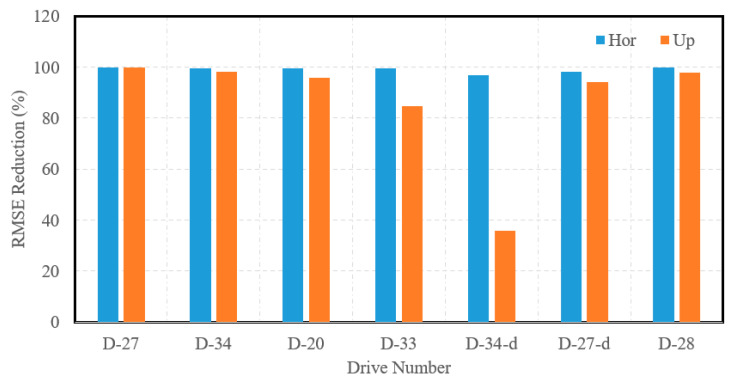
Accuracy improvement expressed by INS RMSE reduction for the horizontal and up directions, raw residential KITTI datasets.

Finally, Figure 7 showcases the RMSE reductions of the INS position estimations for the horizontal and up directions for all drives of the KITTI dataset. It is worth mentioning that the average reduction of INS RMSE for all residential drives was approximately 99% and 86% in the horizontal and up directions, respectively.

### 4.2. Highway Datasets—Case Study 2

**Figure 8:**
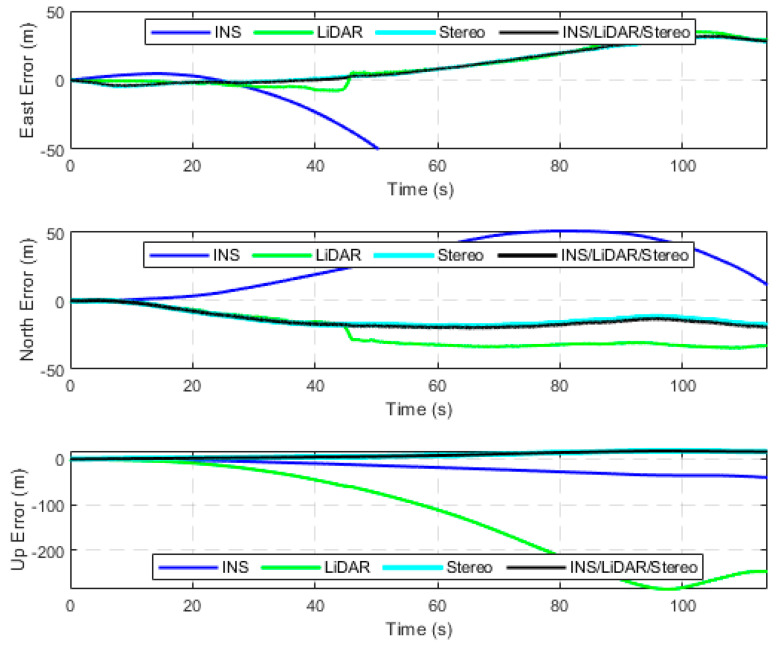
Positional errors expressed in the ENU frame, D-42.

**Figure 9:**
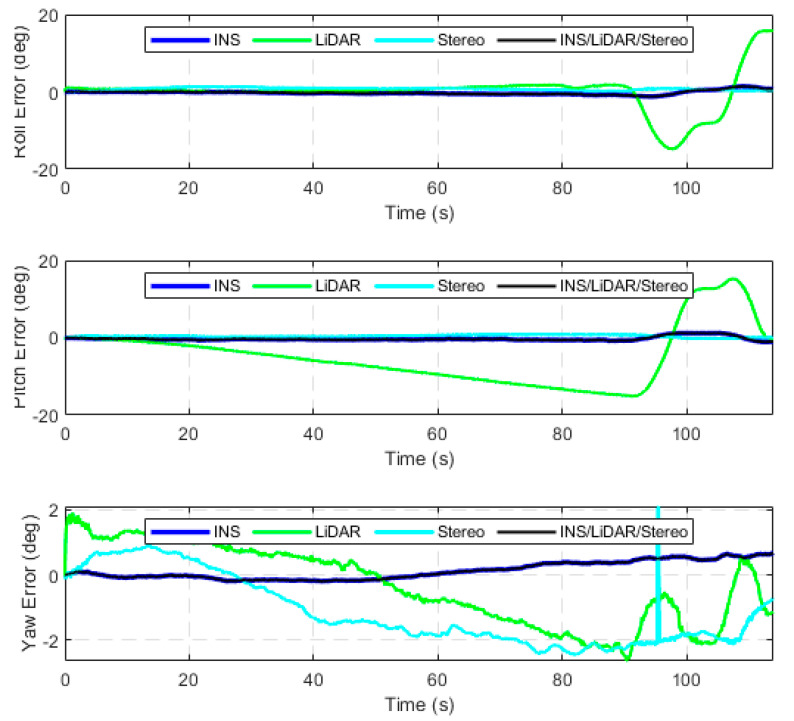
Attitude angles’ errors, D-42.

The second case study involves testing the performance of the developed integrated navigation system using raw highway KITTI datasets. Four highway datasets were considered, which totalled a driving distance and time of roughly 5 kilometres and 5 min, respectively. Detailed results are provided for D-42, which is a 2591-m driving segment with a driving time of 121.19 s. Figure 8 and Figure 9 present the position errors in the ENU frame and attitude errors (roll, pitch, and yaw), respectively. The statistics of these errors are presented in Table 3.

It is noticeable from Figure 8 and Figure 9 that the integrated navigation system persists to have the same behaviour as shown in the first case study. That is, the system follows the position estimations of the more accurate sensors (stereo SLAM) while following the most accurate attitude estimates by the INS.

**Figure 10:**
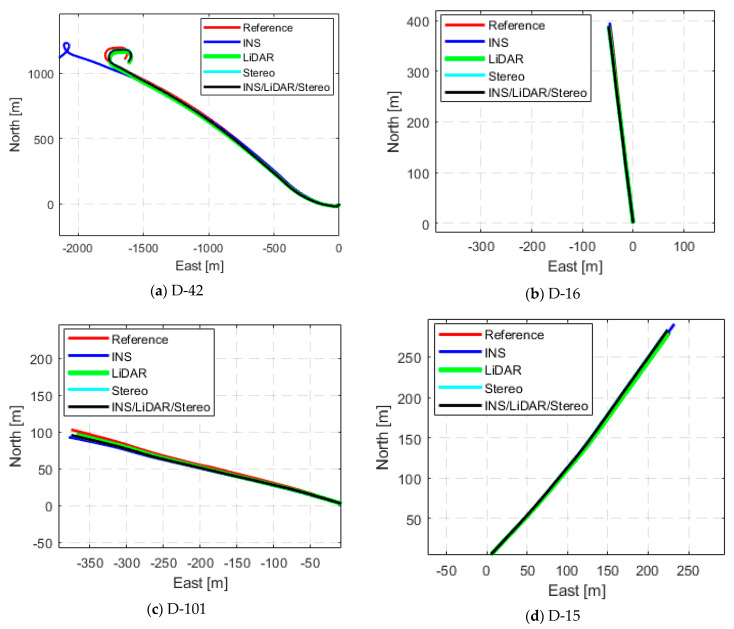
Comparison of trajectories of the raw highway KITTI drives in the ENU frame.

**Figure A11:**
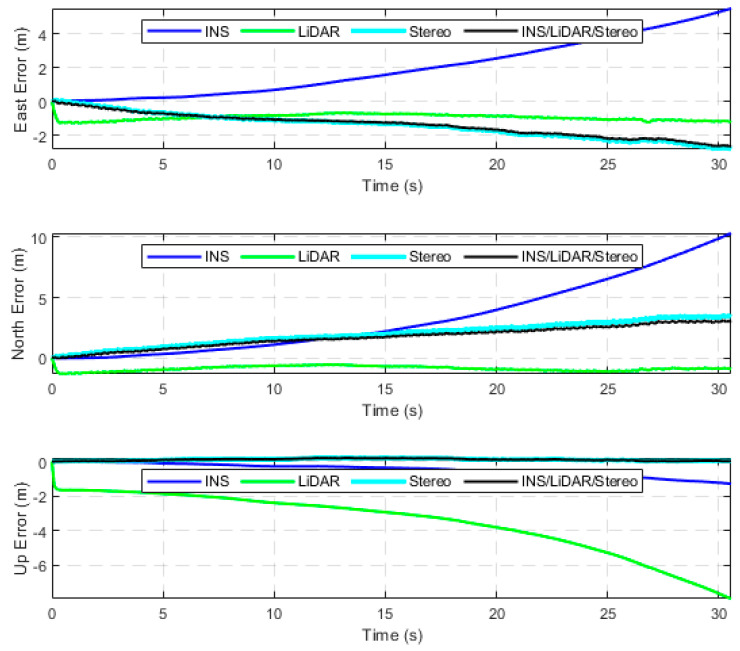
Positional errors expressed in the ENU frame, D-15.

**Figure A12:**
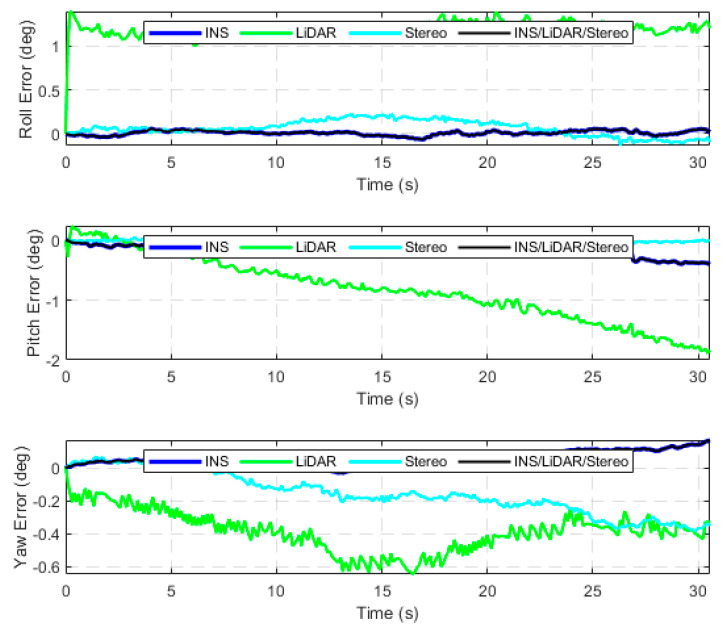
Attitude angles’ errors, D-15.

**Figure A13:**
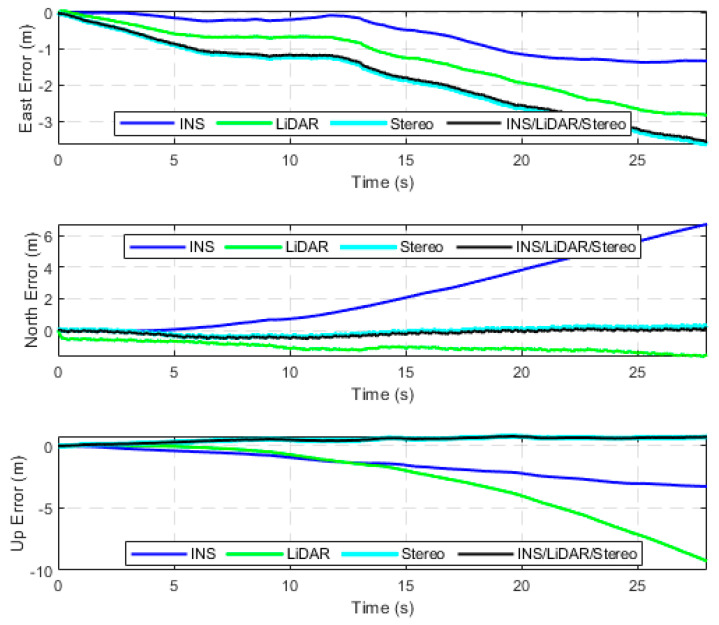
Positional errors expressed in the ENU frame, D-16.

**Figure A14:**
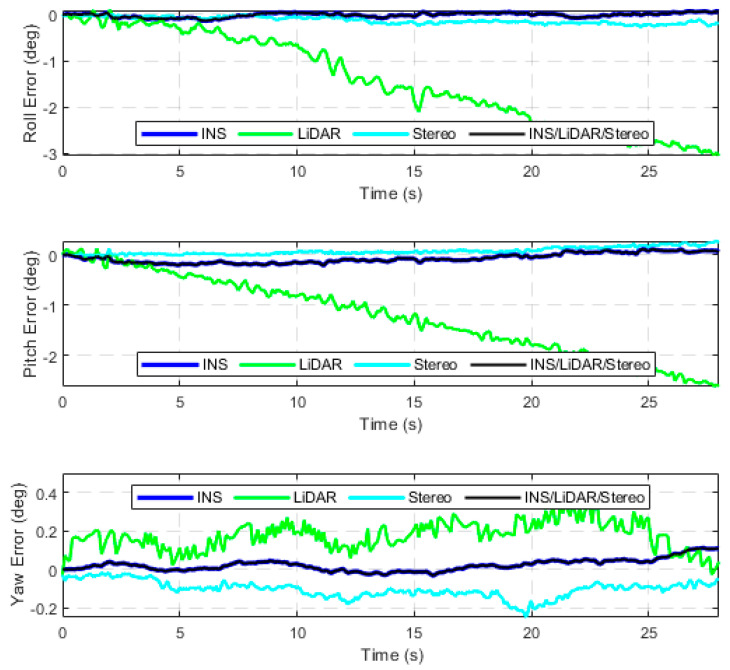
Attitude angles’ errors, D-16.

**Figure A15:**
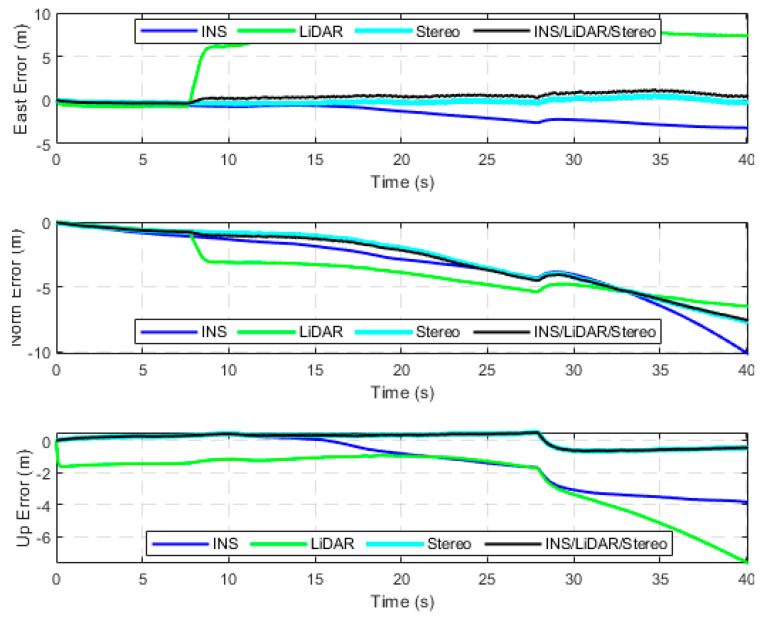
Positional errors expressed in the ENU frame, D-101.

**Figure A16:**
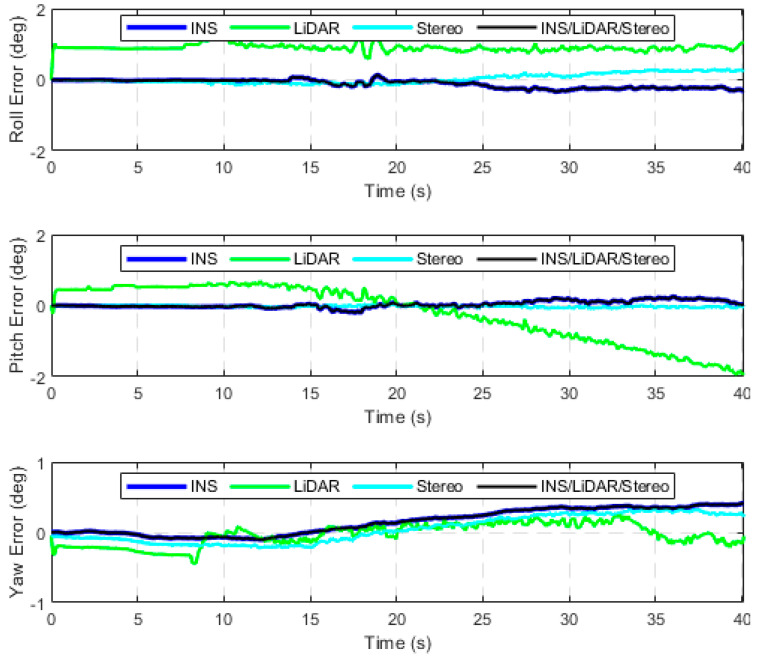
Attitude angles’ errors, D-101.

Having conducted the same analysis for the remaining highway datasets, Figure A11, Figure A12, Figure A13, Figure A14, Figure A15 and Figure A16 in Appendix A provide a detailed comparison of the positional and attitude errors of the datasets. In addition, Figure 10 presents a graphical comparison between the trajectories of the INS, LiDAR SLAM, Stereo SLAM, the integrated navigation system and the ground truth trajectory in the ENU reference frame.

**Figure 11:**
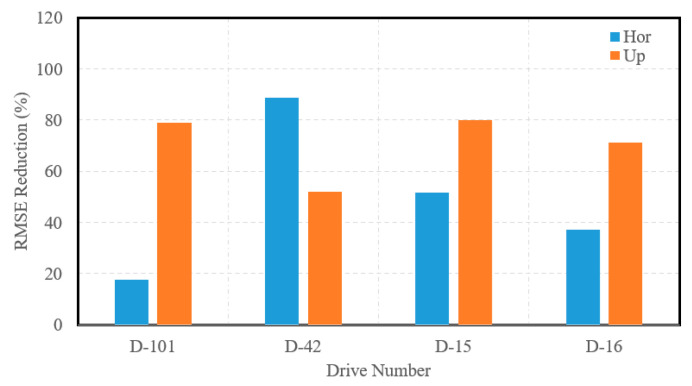
Accuracy improvement expressed by INS RMSE reduction for the horizontal and up directions, raw highway KITTI datasets.

Figure 11 presents the RMSE reductions of the INS position estimations in the horizontal and up directions for highway drives considered in the second case study. It is important to note that, on average, the INS RMSE was reduced by approximately 50% for the horizontal direction and 73% for the up direction in highway drives.

### 4.3. Comparison to State-of-the-Art Models

It is worth mentioning that even though the developed INS/LiDAR/Stereo navigation system does follow the position of the stereo SLAM and the attitude of the INS, the LiDAR plays a vital role as a redundant onboard sensor. If some unexpected malfunction occurs to the stereo camera, the navigation system will continue to operate efficiently to the accuracy of the LiDAR SLAM, which prevents full navigation system failure.

The INS/LiDAR/Stereo navigation system yielded superior performance to the system we developed in [32] as illustrated in Table 4. The table compares both navigation models using a sample of the KITTI dataset. The accuracy improvement in the positional RMSE is around 50% and 64% for the horizontal and up directions, respectively. In addition, it is important to mention that the INS/LiDAR navigation model was compared in [32] to other state-of-the-art SLAM algorithms, which promotes the accuracy of our INS/LiDAR/Stereo system versus both our previous work and other state-of-the-art SLAM algorithms [29,30,41].

## 5. Conclusions

In this research, an integrated INS/LiDAR/Stereo navigation system was developed and tested in various driving scenarios using the raw KITTI dataset. The datasets were broken into two case studies, namely residential and highway datasets. Both case studies covered residential areas driving scenarios at relatively low driving speeds with dense features and driving in highway areas at higher speeds and fewer features. In both case studies, the developed integrated navigation system followed the position of the stereo SLAM estimation, albeit the attitude estimations of the INS. In addition, the integrated system led to an overall improvement in the INS RMSE for all case studies of 83% and 82% in the horizontal and up directions, respectively. Finally, the developed navigation model was superior to our previously developed INS/LiDAR navigation model by an average accuracy improvement of approximately 50% and 64% for the horizontal and up directions, respectively.

## Figures and Tables

**Table 1:** Trajectory information of the raw KITTI drives under consideration [39].

Drive Label	Drive Number	Length (m)	Time (s)	Average Speed (km/h)	No. of Frames
Residential datasets					
D-27	2011_10_03_drive_0027_sync	3669.18	465.97	28.35	4497
D-33	2011_09_30_drive_0033_sync	1709.57	165.31	37.23	1594
D-34-d	2011_09_30_drive_0034_sync	920.52	126.88	26.12	1224
D-34	2011_10_03_drive_0034_sync	5043.67	481.31	37.72	4642
D-20	2011_09_30_drive_0020_sync	1233.74	114.49	38.79	1104
D-27-d	2011_09_30_drive_0027_sync	692.47	114.85	21.71	1106
D-28	2011_09_30_drive_0028_sync	4208.65	537.78	28.17	5177
Highway datasets					
D-42	2011_10_03_drive_0042_sync	2591.80	121.19	76.99	1170
D-15	2011_09_26_drive_0015_sync	362.81	30.65	42.61	297
D-101	2011_09_26_drive_0101_sync	1299.13	96.62	48.40	936
D-16	2011_09_30_drive_0016_sync	404.71	28.84	50.52	278

**Table 2:** Position (m) and attitude angles (deg) error statistics—D-34.

	INS			LiDAR			Stereo			INS/LiDAR/Stereo		
	Mean	RMSE	Max	Mean	RMSE	Max	Mean	RMSE	Max	Mean	RMSE	Max
East	485.60	795.29	1556.31	−5.33	6.46	13.75	−4.66	7.28	16.76	−4.72	6.90	15.00
North	718.27	1840.00	5986.33	−25.25	26.30	36.37	3.73	6.51	12.98	1.09	4.75	8.93
Horizontal	1524.50	2004.51	6046.38	26.05	27.08	37.68	8.67	9.76	17.91	7.34	8.38	15.31
Up	169.47	322.74	1082.86	29.38	33.62	60.78	5.68	6.22	11.05	5.68	6.22	11.04
Roll	0.237	2.022	6.144	0.647	3.111	6.706	0.258	1.051	2.379	0.232	2.020	6.190
Pitch	−0.051	2.655	6.298	−0.420	3.559	6.475	0.123	0.820	2.595	−0.048	2.671	6.350
Yaw	0.932	1.072	4.015	−1.254	1.601	4.383	0.831	0.980	4.880	0.966	1.119	4.083

**Table 3:** Position (m) and attitude angles (deg) error statistics—D-42.

	INS			LiDAR			Stereo			INS/LiDAR/Stereo		
	Mean	RMSE	Max	Mean	RMSE	Max	Mean	RMSE	Max	Mean	RMSE	Max
East	−129.93	194.98	502.57	10.79	17.82	35.49	10.53	16.23	31.39	10.56	16.36	31.65
North	27.09	32.69	50.88	−22.50	25.72	34.33	−13.12	14.29	18.42	−13.98	15.25	19.91
Horizontal	135.94	197.70	502.70	26.86	31.28	48.39	19.32	21.63	34.70	19.90	22.36	35.66
Up	17.93	22.23	40.58	123.55	161.39	285.49	8.73	10.70	17.86	8.73	10.70	17.86
Roll	−0.225	0.557	1.421	0.026	4.820	15.838	0.730	0.797	1.357	−0.224	0.555	1.419
Pitch	−0.266	0.570	1.201	−4.770	8.955	15.270	0.436	0.526	0.916	−0.266	0.570	1.202
Yaw	0.130	0.299	0.682	−0.328	1.260	2.654	−1.144	1.566	2.465	0.129	0.297	0.677

**Table 4:** Comparing the developed model to the INS/LiDAR model developed in [32].

	INS/LiDAR/Stereo, RMSE (m)	INS/LiDAR, RMSE (m)	Accuracy Improvement (%)
	D-28		
Horizontal	8.40	15.06	44.22
Up	7.83	13.57	42.30
	D-42		
Horizontal	22.36	32.15	30.45
Up	10.70	23.58	54.62
	D-101		
Horizontal	3.67	14.82	75.24
Up	0.41	6.03	93.20

## Data Availability

The data used in this study can be found at: http://www.cvlibs.net/datasets/kitti/, accessed 10 March 2023.
